# Electromagnetic field exposure affects the calling song, phonotaxis, and level of biogenic amines in crickets

**DOI:** 10.1007/s11356-023-28981-0

**Published:** 2023-07-28

**Authors:** Joanna Wyszkowska, Jarosław Kobak, Hitoshi Aonuma

**Affiliations:** 1 Research Institute for Electronic Science, Hokkaido University, Sapporo, Hokkaido 060-0812 Japan; 2 Department of Animal Physiology and Neurobiology, Faculty of Biological and Veterinary Sciences, Nicolaus Copernicus University, Lwowska 1, 87-100, Toruń, Poland; 3 Department of Invertebrate Zoology and Parasitology, Faculty of Biological and Veterinary Sciences, Nicolaus Copernicus University, Lwowska 1, 87-100, Toruń, Poland; 4 Graduate School of Science, Kobe University, 1-1 Rokkodai, Nada-Ku, Kobe, Hyogo 657-8501 Japan

**Keywords:** Electromagnetic field, HPLC, Stress, Mating behavior, Communication, Insects

## Abstract

The electromagnetic field (EMF) is ubiquitous in the environment, constituting a well-known but poorly understood stressor. Few studies have been conducted on insect responses to EMF, although they are an excellent experimental model and are of great ecological importance. In our work, we tested the effects of EMF (50 Hz, 7 mT) on the cricket *Gryllus bimaculatus*: the male calling song pattern, female mate choice, and levels of biogenic amines in the brain. Exposure of males to EMF increased the number and shortened the period of chips in their calling song (by 2.7% and 5% relative to the control song, respectively), but not the sound frequency. Aged (3-week-old) females were attracted to both natural and EMF-modified male signals, whereas young (1-week-old, virgin) females responded only to the modified signal, suggesting its higher attractance. Stress response of males to EMF may be responsible for the change in the calling song, as suggested by the changes in the amine levels in their brains: an increase in dopamine (by 50% relative to the control value), tyramine (65%), and serotonin (25%) concentration and a decrease in octopamine level (by 25%). These findings indicate that *G. bimaculatus* responds to EMF, like stressful conditions, which may change the condition and fitness of exposed individuals, disrupt mate selection, and, in consequence, affect the species’ existence.

**Graphical Abstract:**

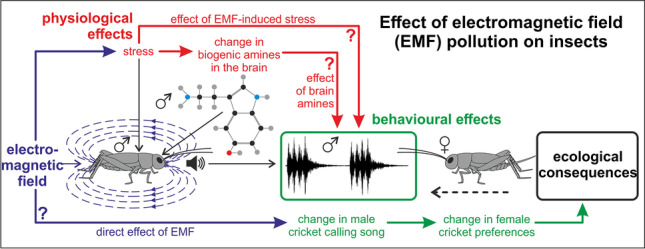

**Supplementary Information:**

The online version contains supplementary material available at 10.1007/s11356-023-28981-0.

## Introduction

The electromagnetic field (EMF) has always been naturally present in the environment*.* However, the increasing number of artificial sources of EMF (e.g., powerlines, wiring, electrical appliances, and wireless communication), with an enormous range of new frequencies, modulations, and intensities, constitutes an emerging source of environmental pollution and raises concern about their unfavorable effects on organisms (Comba and Fazzo 2009; Mattsson and Simkó 2012; WHO 2007). So far, little has been done to consider the potential environmental impacts of EMF, although it seems to represent a potentially significant environmental stressor (Blank and Goodman 2009) that can lead to memory deficits (Jadidi et al. 2007), stress, anxiety (Liu et al. 2008), and depression-like behavior (Szemerszky et al. 2010).

The majority of research in the area concerns mammals, practically neglecting lower organisms, such as insects, which are great models to study the impact of EMF at multiple levels of biological organization, including cognitive, behavioral, ecological, physiological, and molecular effects and their mechanisms (Baier et al. 2002; Horch et al. 2017; Wyszkowska et al. 2018). Moreover, given the great contribution of insects to global biodiversity and ecosystem services, as well as their multiple environmental and economic roles (e.g., as pests, pollinators, food sources, predators, and herbivores), knowledge of their responses to emerging pollution sources is crucial for understanding the functioning of contemporary ecosystems, identifying potential economic threats, and designing novel measures of pest control and biodiversity conservation (Schowalter et al. 2018). Recently, EMF has been pointed out as one of the reasons for the rapid decline of the honeybee, known as an important pollinator of global importance. Current trends show substantial declines in insects in general for at least the last 3 decades (Hallmann et al. 2017; Leather 2018).

One of the mechanisms of the negative effects of EMF on organisms can be the disruption of mating behavior, including sending and receiving sexual signals and finding suitable partners. By assessing species-specific sexual signals, individuals make informed decisions about optimal mate selection, resulting in reproductive fitness benefits. However, these communication systems are susceptible to interference from human-made factors present in the environment, leading to their disruption and fitness reduction due to inefficient mate choice.

In the present study, we analyzed the effect of EMF (50 Hz, 7 mT) on the reproductive behavior of the two-spotted cricket, *Gryllus bimaculatus*. This species is an important model organism in neurobiological, physiological, developmental, and regeneration research (Horch et al. 2017; Mito and Noji 2008). Its mating behavior is well understood and described: males rub their forewings together (Baker et al. 2019), generating species-specific acoustic signals known as calling songs used to communicate information critical for reproduction (location and quality of the male) to females.

We hypothesized that exposure to EMF would affect the quality of male calling songs and, in consequence, their attractiveness to females. Moreover, we assumed that EMF would cause changes in the levels of biogenic amines in the insect brain, indicating increased stress.

## Material and methods

### Animals

Crickets, *Gryllus bimaculatus* (De Geer), were raised in a laboratory colony on a 14 h:10 h light and dark cycle (lights on at 6:00 h) at 28 ± 2 °C. They were fed with insect food (Sankyo Lab, Tokyo, Japan) and water ad libitum. Each developmental stage was kept in a plastic box (300 mm × 500 mm × 250 mm). Newly emerged virgin females and males were collected from the culture every day to create cohorts of the same age. Insects were kept in single- and mixed-sex cultures. After sexual maturation, 1- and 3-week-old crickets were used in experiments.

### Electromagnetic field exposure system

**Fig. 1:**
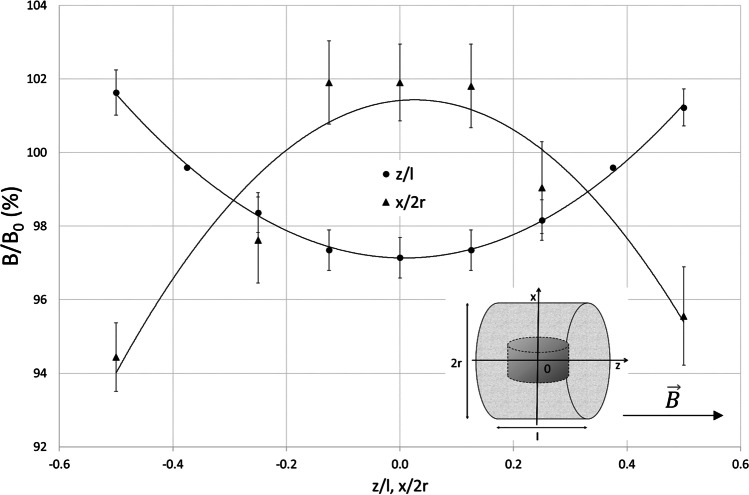
The distribution of the electromagnetic field along the main axis of the coil within the area of the animal chamber and the location of the exposure chamber within the coil and coordinate system used for the plot. $\overrightarrow{\mathrm{B}}$ is the magnetic flux density vector and *B*/*B*_0_ is the normalized magnetic flux density relative to the center point of the coil, where *B* is the 7 mT, *z*/*l* is the normalized distance from the coil center along the *z*-axis, *x*/2*r* is the normalized distance from the solenoid center along the *x*-axis, *l* is the coil length, 21 cm, and 2*r* is the coil inner diameter, 19 cm

The electromagnetic field (EMF) with the domination of magnetic component was generated by a 19 cm-diameter coil designed by EiE (Elektronika & Elektromedycyna Sp. J., Otwock, Poland). This exposure system has been described in detail previously (Bieńkowski and Wyszkowska 2014; Trawiński et al. 2010). The coil generated a homogeneous, sine-wave alternating EMF at 50 Hz. The distribution of magnetic flux density within the coil along the *Z* and *X* axes is shown in Fig. 1. The non-homogeneity of the field within the area containing the animal chamber was approximately 10%. The magnetic field strength was controlled before each experiment using a Gaussmeter (Model GM2, AlphaLab, Inc., USA). Animals were exposed to EMF (50 Hz, 7 mT) in a glass exposure chamber (diameter: 10 cm, height: 7.5 cm) closed with fly screen mesh and located within the coil. The temperature in the exposure chamber was set to 24 ± 1 °C and monitored using thermocouples. The relative humidity inside the chamber during EMF exposure was 43%.

### Calling song recording and analysis

**Fig. 2:**
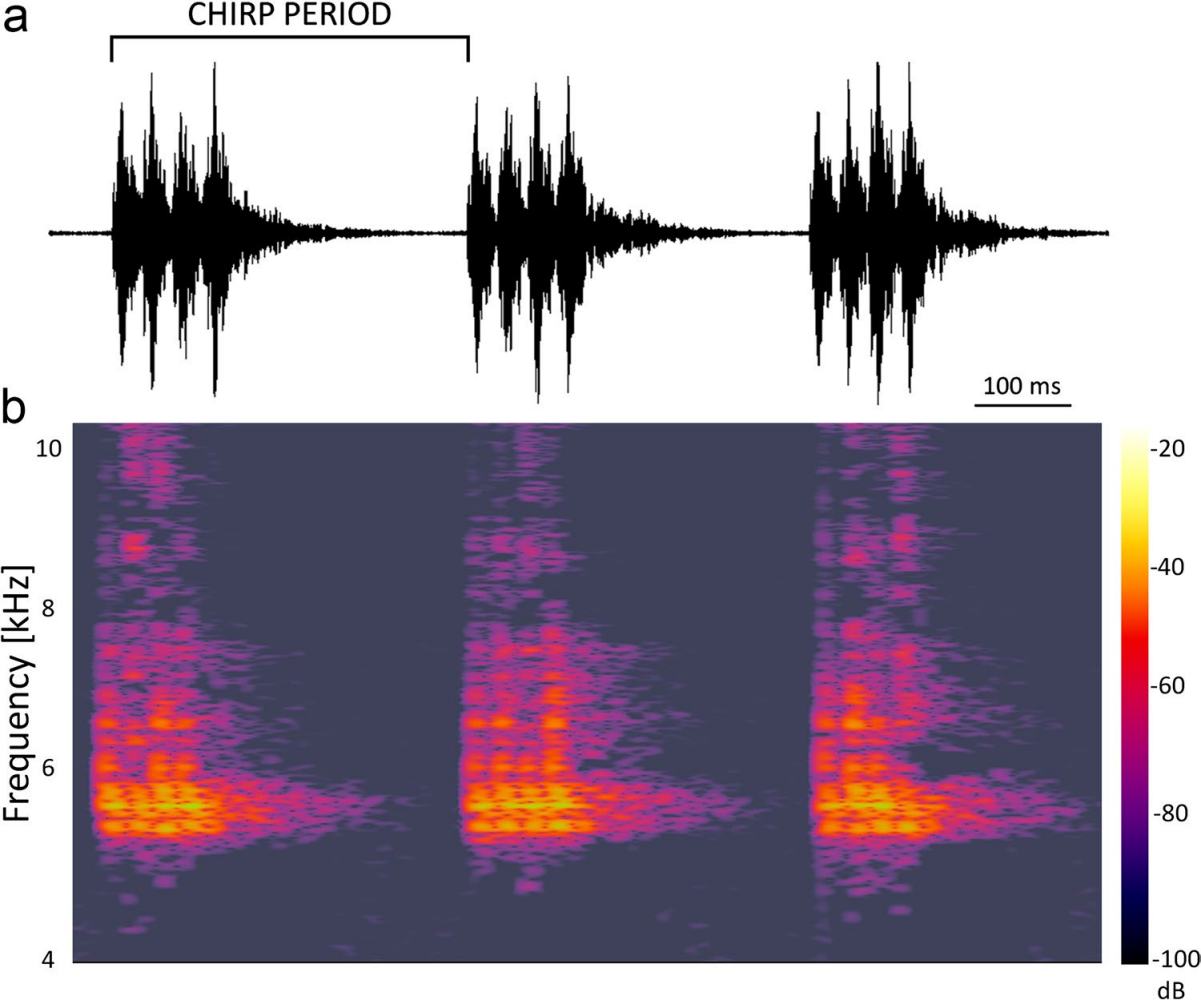
Chirp sequence in the male calling song of *Gryllus bimaculatus*. **a** Oscillogram of chirps comprising the song with time-aligned spectrogram **b** generated in Audacity 3.2.1. The colors represent the decibel levels for a specific frequency at a specific time point. Brighter colors mean stronger intensities. The decibel values range from − 20 (loudest) down to − 100 dB (softest)

Individual calling songs of males were recorded digitally at a constant temperature (24 ± 1 °C) using a portable audio recorder: a linear PCM recorder, model PCM-D50 (Sony Corporation, Tokyo, Japan). Recordings were made during the first 3 h of the dark cycle. A female, selected randomly from the bulk stock, was put together with a male in an exposure chamber. The exposure chamber with the insects was located inside the coil. The exposure lasted for 10 min and consisted of the initial period before the EMF was turned on (3 min), the actual exposure to the EMF (4 min), and the post-exposure period after the EMF was turned off (3 min). As soon as the male began to court the female, the audio was recorded. Recordings were obtained for 15 males and included three 1-min measurement periods: (1) “before” (2nd–3rd min of the exposure), (2) “during” (0.5–1.5 min after the EMF was turned on), and (3) “after” (2nd–3rd min after the EMF was turned off) the exposure to the EMF (Fig. S1). The song recordings were analyzed using WavePad software ver. 10.26 (NCH Software, CO, USA) and freeware Audacity 3.2.1 (https://www.audacityteam.org/). Parameters measured during each 1-min measurement period of the calling song were (1) the chirp period (Fig. 2): a mean of 10 random chirps; (2) chirp rate: the number of chirps per min measured directly in oscillograms; and (3) the dominant frequency (highest peak) (Hz) obtained from the spectrum using the fast Fourier transform.

### Phonotaxis experiment

Male calling songs were recorded by exposing an animal in the exposure chamber for 5 min to the EMF and then for another 5 min with the EMF turned off. To test the responses of females to natural and EMF-modified male signals, male calling songs were obtained by putting a single male in the exposure chamber in the presence of a female and recording the male song for 5 min with the EMF turned on and then for another 5 min with the EMF turned off.

**Fig. 3:**
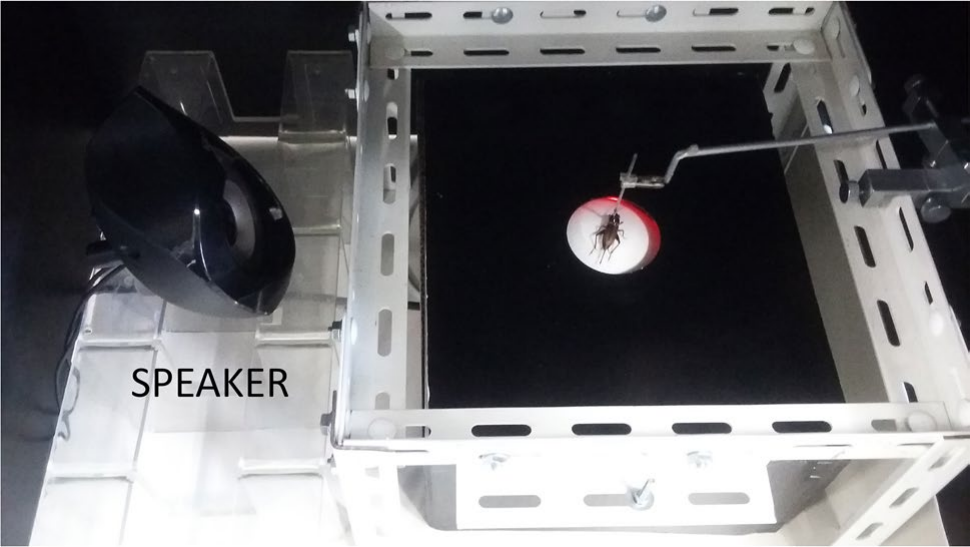
Phonotaxis trackball system. A female cricket (*G. bimaculatus*) was tethered at the pronotum and placed on a trackball suspended above the arena (30 × 30 cm). The speaker was used for acoustic stimulation

A single female cricket was positioned on the top of a styrofoam trackball (φ = 10 cm, 8.7 g) in its natural walking posture (Fig. 3). The tip of a wire holder was fixed to the center of the pronotum with a mixture of beeswax and resin to keep the animal in place. The walking insect moved in place, changing the position of the trackball (Hedwig 2017). The motion of the trackball was recorded using optical motion sensors repurposed from a computer mouse (Taylor et al. 2015). Tracking data were collected and processed with the custom-made software that kept track of changes in the *x* and *y* coordinates as the ball rotated in response to the insect movements (Owaki et al. 2021). The trackball movements allowed us to determine the insect’s speed and walking direction (Hedwig 2017).

Experiments were conducted on females responding to a male calling song played from a loudspeaker situated on the left side of the experimental arena (Fig. 3). The sound pressure was set at 85 dB, which has been commonly used in similar behavioral experiments (e.g., Choi et al. 2012). Females were divided into two groups: 3 weeks old (which had contact with males before in a mixed-sex colony, *N* = 16) and 1-week-old (virgins, no previous contact with males, *N* = 16) and placed individually on the ball. They were acclimated to the experimental set-up for 3 min prior to the experiment. Then, all individuals were exposed to the “control” signal (a calling song recorded under control conditions) for 5 min. Then, after a 5-min interval, the females from the experimental group (*N* = 8 per each age group) were exposed to the “changed” signal (a calling song of a male exposed to EMF) for 5 min, whereas the control females (*N* = 8 per each age group) were again exposed to the “control” signal. All trials were performed in darkness at 24 ± 1 °C.

### Phonotaxis data analysis

The entire recording (two 5-min periods) was divided into 5-s intervals. For each interval, using the *xy* coordinates describing the cricket position, we determined (1) the angle between the direction of translocation and the direction straight towards the signal source and (2) the translocation distance. The angles ranged from 0 to 360°, with values close to 0 and 360° pointing towards the signal, those approaching 180° pointing away from the signal, and those around 90 or 270° perpendicular to the signal direction. As we were only interested in the axis parallel to the signal direction (i.e., it did not matter whether a cricket deviated left or right from this direction), we transformed the angle values (*D*) according to the following formula:

$${D}{\prime}=\left\{\begin{array}{c}90^\circ -D for D\le 180^\circ \\ D-270^\circ for D>180^\circ \end{array}\right.$$

The transformed variable ranged from − 90 to 90°, direction towards the signal being 90°, direction away from the signal being − 90°, and direction perpendicular to the signal position being 0°. Thus, a circular angle variable was transformed into a linear variable that could be analyzed using standard statistical methods. Then, for each of the two 5-min periods, we calculated (1) the mean direction (angle, *D*′), (2) the difference between the total distances moved towards and away from the signal (including only movements with directions higher than 45° or lower than − 45°), and (3) the difference between the total times spent on moving towards and away from the signal (again, only when the modulus of the direction > 45°). The latter two variables were calculated according to the following formula:

$$\mathrm{\Delta M}= \left({M}_{T}-{M}_{A}\right)/\left({M}_{T}+{M}_{A}\right)$$

where Δ*M* is the difference in movement time/distance between both directions and *M*_*T*_ and *M*_*A*_ are the movement time/distance towards and away from the signal, respectively. These variables ranged from − 1 to 1, indicating the prevalence of movement away (− 1), irrespective of (0), and towards (1) the signal.

### Biogenic amine levels

The levels of serotonin (5-HT), dopamine (DA), octopamine (OA), and tyramine (TA) were measured in male crickets randomly divided into two groups: individuals exposed individually to the EMF in the exposure chambers (see above) for 10 min (*N* = 10) and control individuals (*N* = 8). The control insects were tested under the same experimental conditions but in the absence of EMF (the ambient magnetic field strength < 10 µT).

Immediately after the exposure, crickets were frozen using liquid N_2_. Their brains were dissected out of the ice-cold cricket physiological saline (140 mM NaCl, 10 mM KCl, 1.6 mM CaCl_2_, 2 mM MgCl_2_, 44 mM glucose, 2 mM TES, pH 7.2), put separately into glass homogenizers, and homogenized in 50 µl of ice-cold 0.1 M perchloric acid containing 5 ng of 3,4-dihydroxybenzylamine (DHBA, SIGMA, St Louis, MO, USA) as an internal standard. After centrifugation of the homogenate (0 °C, 15,000 rpm, 30 min), 40 µl of the supernatant was collected. Biogenic amines in each sample were measured using high-performance liquid chromatography (HPLC) with electrochemical detection (ECD) (Aonuma 2020; Aonuma and Watanabe 2012; Owaki et al. 2021; Wada-Katsumata et al. 2011). The HPLC-ECD system was composed of a pump (EP-300, EICOM Co., Kyoto, Japan), an auto-sample injector (M-510, EICOM Co., Kyoto, Japan), and a C18 reversed-phase column (250 mm × 4.6 mm internal diameter, 5 μm average particle size, CAPCELL PAK C18MG, Shiseido, Tokyo, Japan) heated to 30 °C in a column oven. A glass carbon electrode (WE-GC, EICOM Co.) was used for electrochemical detection (ECD-100, EICOM Co.). The detector potential was set at 880 mV versus an Ag/AgCl reference electrode, also maintained at 30 °C in a column oven. The mobile phase containing 0.18 M chloroacetic acid and 16 µM disodium EDTA was adjusted to pH 3.6 with NaOH. Sodium-1-octanesulfonate at 1.85 mM as an ion-pair reagent and CH_3_CN at 8.40% (*v*/*v*) as an organic modifier were added to the mobile phase solution. The flow rate was kept at 0.7 ml/min. The chromatographs were acquired using the computer program PowerChrom (eDAQ Pty Ltd., Denistone East, NSW, Australia). The supernatants of the samples were injected directly into the HPLC column. After the acquisition, they were processed to obtain the level of biogenic amines in the same sample by the ratio of the peak area of the substances to the internal standard DHBA (3,4-dihydroxybenzylamine, SIGMA).

### Statistical analysis

Data were analyzed using IBM SPSS Statistics for Windows, Version 25.0. Armonk (IBM Corp., Released 2017, NY: IBM Corp.) and GraphPad Prism version 7.00 for Windows (GraphPad Software, La Jolla, CA, USA). All data were tested for normality (Shapiro’s test) and homogeneity of variance (Levene’s test).

A principal component analysis (PCA) (correlation matrix, varimax rotation with the Kaiser normalization) was applied to the variables characterizing the recorded songs (chirp period, chirp rate, dominating frequency). The obtained principal components were analyzed using 1-way repeated measures ANOVAs to check for differences between the three parts of the song generated by each male individual. Significant ANOVA effects were further evaluated with sequential Bonferroni-corrected *t*-tests for paired data.

As the female phonotaxis data were strongly heteroscedastic, we applied nonparametric tests to compare the experimental groups: pairwise Mann–Whitney’s tests to compare the age groups and the control vs. changed signal treatments, as well as Wilcoxon’s signed-rank tests to compare the first vs. second 5-min observation period. Furthermore, we applied one-sample *t*-tests (for which the homoscedasticity assumption does not apply) to compare the mean values of the response variables for each experimental group and period against a theoretical value of 0, indicating no reaction to the signal. The results were sequential Bonferroni corrected for multiple comparisons within each group of tests.

A PCA (correlation matrix, varimax rotation with the Kaiser normalization) was applied to the biogenic amine levels (5-HT, DA, OA, TA) in the brain. The obtained principal components were compared between the control and EMF-exposed individuals using sequential Bonferroni-corrected *t*-tests.

## Results

### Characteristics of male calling songs

**Fig. 4:**
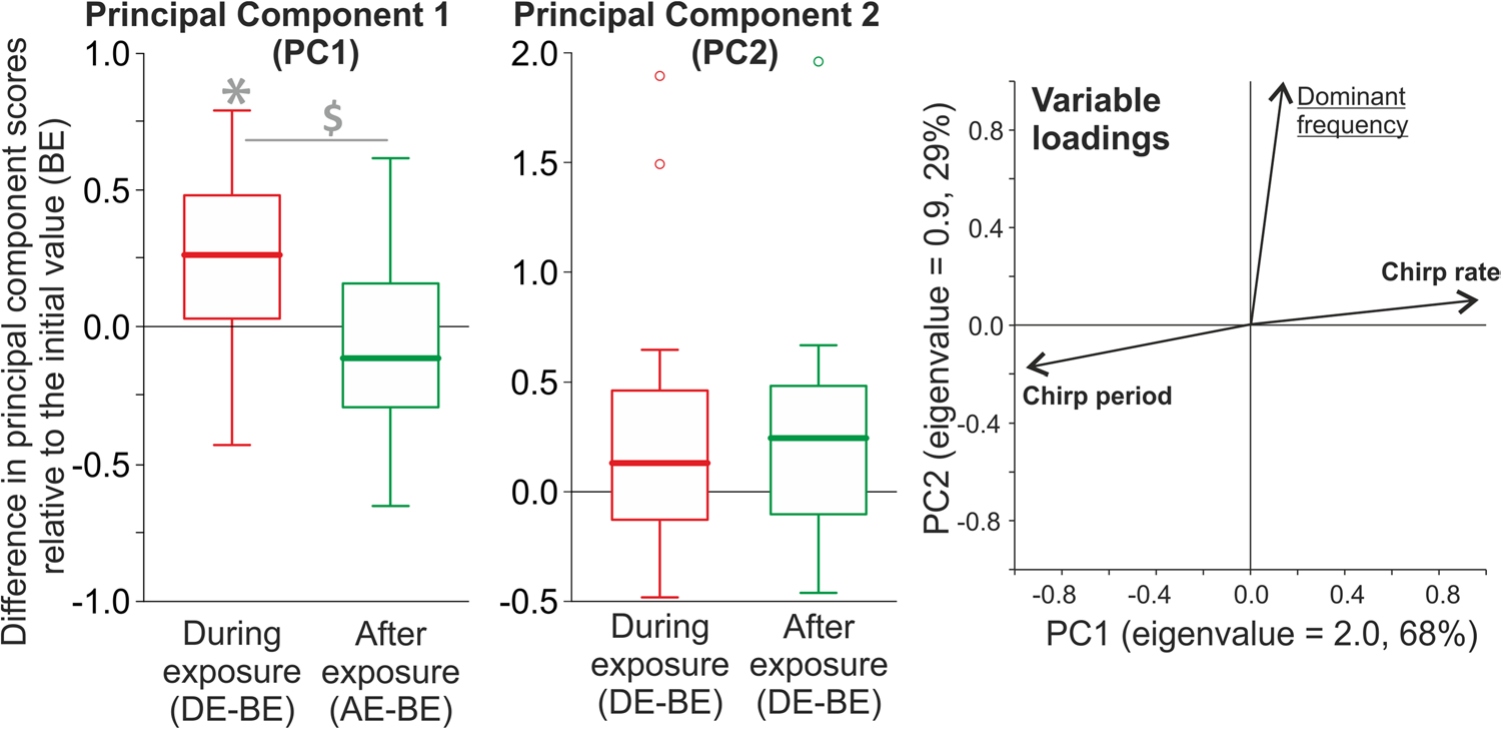
Effect of the electromagnetic field on the male cricket calling song. Right panel: dimension reduction with the principal component analysis (PCA). Eigenvalues (variances explained by particular principal components, PC1 and 2) are reported with percentages of the total variance explained as axis labels. Variable loadings (correlations of the measured variables with particular components) are shown as vectors; variables labelled with bold or underlined font strongly contributed (absolute loading values > 0.5) to PC1 and 2, respectively. The PCA extracted two principal components: PC1 (associated with the chirp rate and negatively correlated with the chirp period) and PC2 (associated with the dominant signal frequency). The PC1 and PC2 scores were obtained for animals before (BE), during (DE), and after (AE) their exposure to EMF. The PC1 and PC2 scores are presented in the left and middle panels, respectively, as differences relative to the initial values (i.e., DE-BE and AE-BE, respectively). Asterisks indicate significant differences between BE and the later periods, and the dollar marks indicate differences between DE and AE. Values above and below 0 indicate an increase and decrease in the PC score compared to initial (BE) values, respectively. The boxplots present medians (horizontal lines), 1st–3rd quartiles (boxes), 1.5*interquartile range (whiskers), and outliers (circles). Raw data for particular signal parameters are shown in Fig. S2)

The PCA extracted two principal components (PC1 and PC2) (Fig. 4). PC1 was positively associated with the chirp rate and negatively correlated with the chirp period, whereas PC2 depended on the value of the dominant frequency. PC1 scores differed among the song parts (repeated measures ANOVA: *F*_2, 28_ = 5.3, *P* = 0.011), with the values obtained during the exposure to EMF significantly larger than those obtained before and after the exposure (Fig. 4). The chirp rate during the exposure to EMF (182.7 ± 7.128 chirps/min) was higher by 2.7% and 4.7% than before (178.7 ± 8.111 chirps/min) and after (174.2 ± 7.706 chirps/min) the exposure, respectively (Fig. S2). The chirp period during the exposure to EMF (331 ± 11.1 ms) was shorter by 5.1% and 3.7% than before (350.8 ± 14.48 ms) and after (343.7 ± 13.41 ms) the exposure (Fig. S2), respectively. No significant differences among the song parts were found for PC2 scores (repeated measures ANOVA: *F*_2, 28_ = 2.31, *P* = 0.118). The spectrograms of calling songs showed high-energy peaks around 5.5 kHz (Fig. 2).

### Phonotaxis of females

**Fig. 5:**
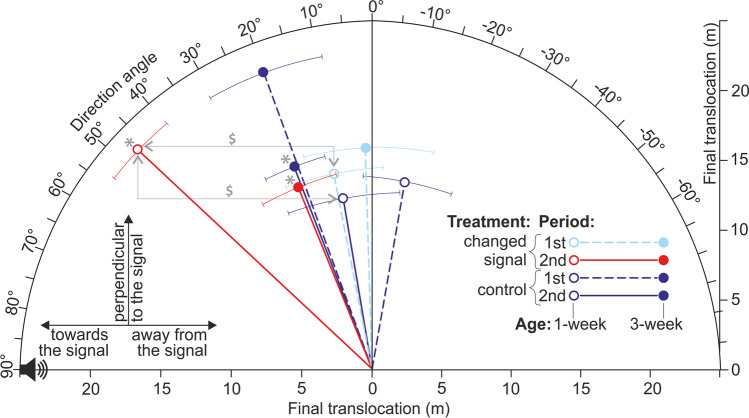
Mean directions (± SE) of movement shown by female crickets of different ages responding to the control signals and signals changed by the electromagnetic field. Directions to the left, right, and top of the plot indicate net movements towards, away from, and perpendicular to the signal source, respectively. Vector lengths reflect the mean final translocations. Dashed and solid lines indicate the first (always the control signal) and second (the control or changed signal, depending on the treatment) periods of the exposure, respectively. Red and blue colors indicate the responses to the changed and control signals, respectively. Open and filled circles indicate 1- and 3-week-old individuals, respectively. Asterisks show significant directional net movements (departures of direction angles from the neutral values of 0), and dollar symbols show significant differences between the treatments and exposure periods

**Fig. 6:**
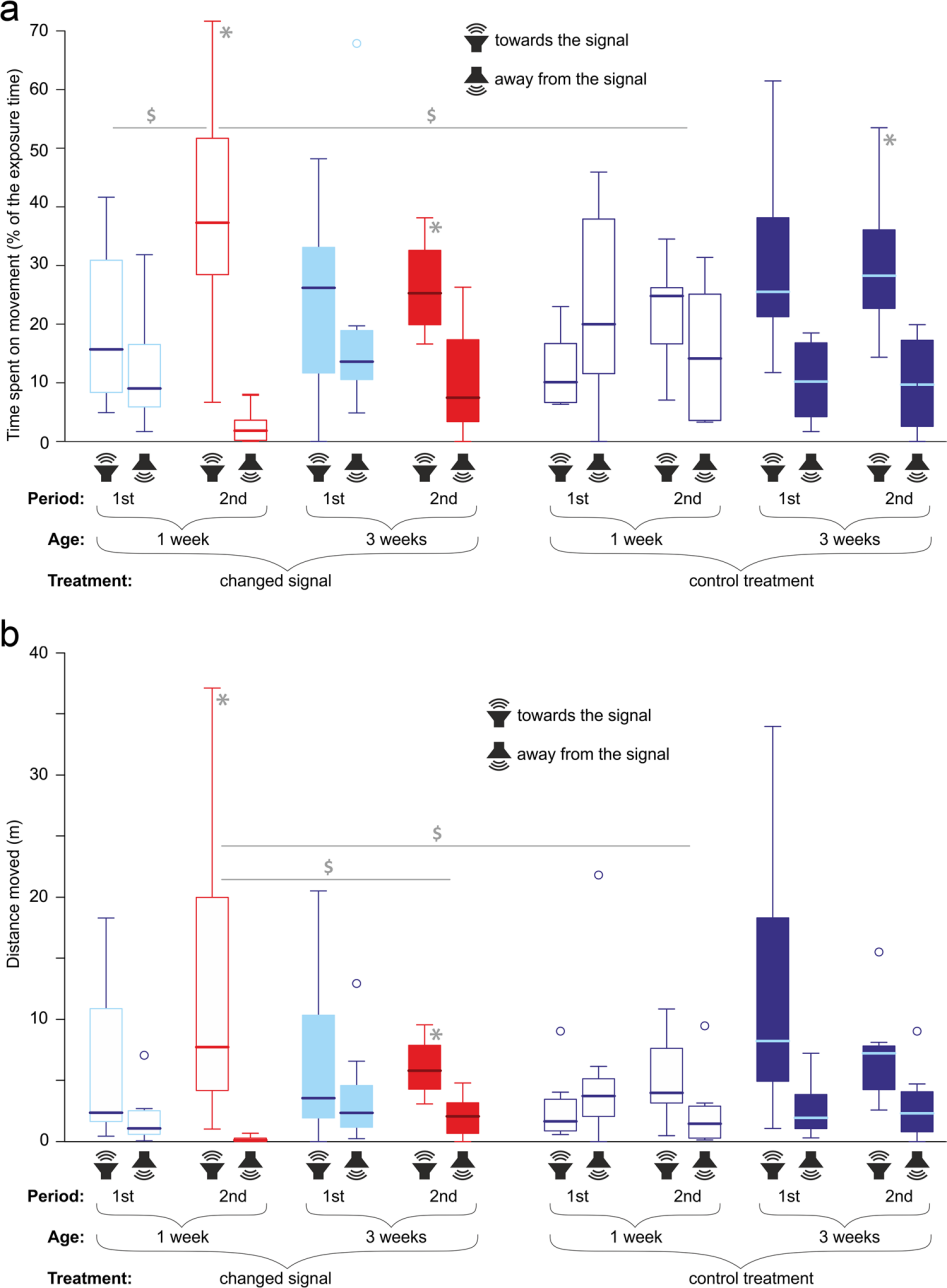
Behavior of female crickets. Times (**a**) and distances (**b**) moved towards and away from the signal by crickets of different ages in response to the control signals and signals changed by the electromagnetic field. Periods: 1st, the first 5-min period (always the control signal); 2nd, the second 5-min period (the control or changed signal, depending on the treatment). Red and blue colors indicate changed and control signals, respectively. Asterisks indicate significant directional movements (departures of differences between both directions from the neutral values of 0), and dollar symbols indicate significant differences between the treatments and exposure periods. The boxplots present medians (horizontal lines), 1st–3rd quartiles (boxes), 1.5*interquartile range (whiskers), and outliers (circles)

In the second observation period, the movement direction angle of females exposed to the EMF-modified signal (both age groups) and to the control signal (only 3-week-old individuals) departed significantly from the neutral 0 value towards the signal source (Fig. 5, Table 1 part a). Moreover, in the second observation period, the times spent on movement (Fig. 6a) and distances moved (Fig. 6b) towards the signal were significantly higher than those moved away from the signal (movement time: all three abovementioned groups, movement distance: both age groups exposed to the changed signal, Table 1 part a). Anyway, it should be noted that a tendency to move directionally towards the signal, though not always significant, was shown by all the females except the control 1-week-old individuals in the first observation period (Figs. 5 and 6).

**Table 1:** Behaviour of female crickets (1 or 3 weeks old) responding to control male signals and signals changed by the electromagnetic field

			Movement angle		Net movement time		Net distance	
Treatment	Age	Period	*t*/*Z*	*P*	*t*/*Z*	*P*	*t*/*Z*	*P*
a. Comparisons with a theoretical value of 0 (one-sample *t*-tests)								
Changed	1 w	1st	1.41	0.202	1.31	0.230	1.87	0.103
Changed	1 w	2nd	8.85	< 0.001*	15.32	< 0.001*	41.72	< 0.001*
Changed	3 w	1st	0.17	0.867	0.66	0.529	0.86	0.417
Changed	3 w	2nd	3.67	0.008*	3.70	0.008*	5.21	0.001*
Control	1 w	1st	1.38	0.210	0.59	0.576	0.58	0.579
Control	1 w	2nd	1.07	0.318	1.29	0.237	1.43	0.196
Control	3 w	1st	2.36	0.050	3.00	0.020	2.32	0.053
Control	3 w	2nd	4.27	0.004*	4.89	0.002*	2.49	0.042
b. Comparisons between the first and second observation periods (Wilcoxon tests)								
Changed	1 w		− 2.52	0.012*	− 2.52	0.012*	− 2.38	0.017
Changed	3 w		− 0.98	0.327	− 0.98	0.327	− 0.70	0.484
Control	1 w		− 2.24	0.025	− 1.68	0.093	− 1.40	0.161
Control	3 w		0.00	1.000	− 0.84	0.401	− 0.14	0.889
c. Comparisons between treatments (Mann–Whitney tests)								
	1 w	1st	− 2.10	0.036	− 1.26	0.208	− 1.58	0.115
	1 w	2nd	− 2.94	0.003*	− 2.74	0.006*	− 2.74	0.006*
	3 w	1st	− 1.37	0.172	− 1.16	0.248	− 0.63	0.529
	3 w	2nd	0.00	1.000	− 0.21	0.833	− 0.42	0.674
d. Comparisons between cricket ages (Mann–Whitney tests)								
Changed		1st	− 0.53	0.600	− 0.32	0.753	− 0.32	0.753
Changed		2nd	− 2.42	0.016	− 2.27	0.023	− 2.59	0.010*
Control		1st	− 2.10	0.036	− 1.58	0.115	− 1.79	0.074
Control		2nd	− 0.84	0.401	− 0.95	0.344	− 0.63	0.528

Treatments: changed–changed signal treatment, control–control treatment. Ages: 1 w, 1 week; 3 w, 3 weeks. Periods: 1st, the first 5-min period (the control signal for all individuals); 2nd, the second 5-min period (the changed or control signal, depending on the treatment). Net movement time/distance – relative difference between times/distances moved towards vs. away from the signal

Asterisks indicate significant differences after applying the sequential Bonferroni corrections for multiple comparisons

The 1-week-old females exposed to the changed signal during the second observation period exhibited more directional movements (Fig. 5) and spent relatively more time moving towards the signal (Fig. 6a) compared to the first observation period (Table 1 part b). Moreover, in the second observation period, the 1-week-old females exposed to the changed signal moved more directionally (Fig. 5), showed higher net movement time (Fig. 6a), and moved a longer net distance towards the signal (Fig. 6b) than the 1-week-old control individuals (Table 1 part c). Finally, in the second observation period, the 1-week-old females exposed to the changed signal exhibited a longer net distance moved in the signal direction (Fig. 6b) than the 3-week-old individuals exposed to the changed signal (Table 1 part d).

### Amine levels

**Fig. 7:**
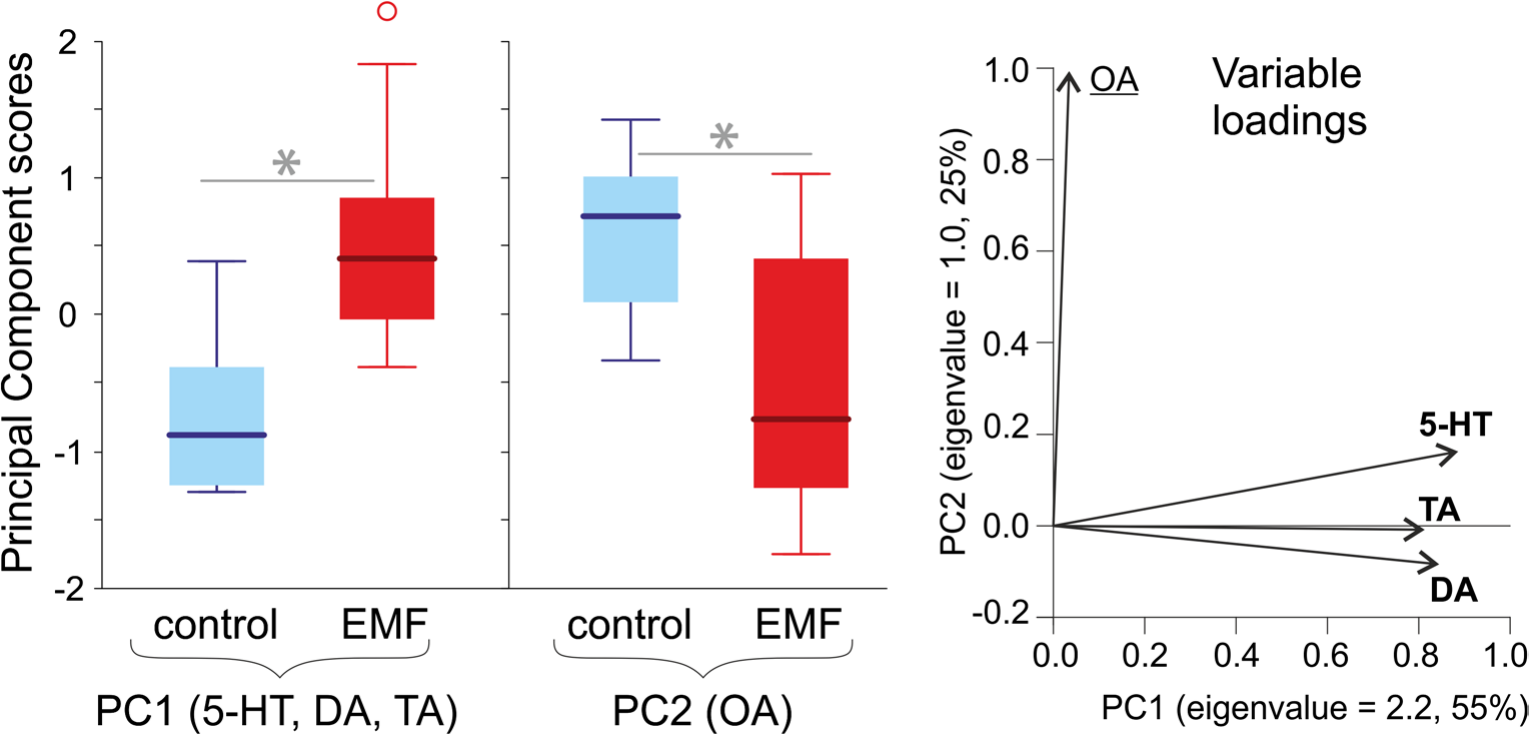
Biogenic amine (5-HT, serotonin; DA, dopamine; OA, octopamine; TA, tyramine) levels in the brains of male crickets exposed to electromagnetic field (EMF) and of control individuals. Right panel: dimension reduction with the principal component analysis (PCA). Eigenvalues (variances explained by particular principal components, PC1 and 2) are reported with percentages of the total variance explained as axis labels. Variable loadings (correlations of the measured variables with particular components) are shown as vectors. Variables labelled with bold or underlined font strongly contributed (absolute loading values > 0.5) to PC1 and 2, respectively. The PCA extracted two principal components: PC1 (associated with 5-HT, TA, and DA) and PC2 (associated with OA). Left panel: principal component scores obtained for the control and EMF-exposed crickets. The boxplots present medians (horizontal lines), 1st–3rd quartiles (boxes), 1.5*interquartile range (whiskers), and outliers (circles). Asterisks indicate significant effects of EMF. Raw data for particular amine levels are shown in Fig. S3

The PCA extracted two principal components (PC1 and PC2). PC1 was associated with the levels of 5-HT, DA, and TA, whereas PC2 was associated with the level of OA (Fig. 7). The scores of PC1 and PC2 differed among the individuals exposed to EMF and control animals (*t*-tests: *t*_16_ = 3.38, *P* = 0.002, *t*_16_ = 2.26, *P* = 0.019, respectively). The crickets exposed to EMF had higher levels of amines associated with PC1: 5-HT (by 25% relative to the control), DA (by 50%), and TA (by 65%), as well as lower levels of OA (by 25%) associated with PC2, compared to the control animals (Fig. 7, Fig. S3).

## Discussion

In this work, we assessed changes in spectral, temporal, and functional features of the male calling song of *Gryllus bimaculatus* due to exposure to EMF*.* In our experiments, we used the EMF with parameters (50 Hz, 7 mT) known for their biological effects, including modification of motor activity (Pešić et al. 2004), induction of depression-like behavior and corticosterone secretion (Kitaoka et al. 2013), free radical generation in the brain (Ciejka et al. 2011), modulation of the antioxidative defense and some of the fitness-related traits (Todorović et al. 2012), and impairment of spatial memory (Jadidi et al. 2007). Additionally, European Union Directive 2013/35/EU (Directive 2013) indicated that EMF of 50 Hz and > 6 mT caused measurable biological effects. Under natural conditions, flying insects can be exposed to EMF of such intensity, e.g., bees flying at a distance of 1 cm from a powerline conductor (Petrović et al. 2013). Moreover, high-voltage power lines emitting EMF are often in the trajectory of many species (i.e., insects and birds). Nevertheless, even much lower intensities of EMF give rise to signals that can affect animal behavior thousands of kilometers away from the source; e.g., EMF caused by storms increased the take‐off rate of locusts that initiated flights (Bergh 1979). Also, exposure to EMF of 20 μT at 50 Hz reduced the olfactory learning of bees (Shepherd et al. 2018). Our study can be a good reference for further analysis of how high levels of EMF might impact other insects providing valuable ecosystem services. Moreover, the responses of insects can be extrapolated to estimate the risks and benefits of human treatments. EMF with similar parameters as those used in our study is commonly applied in magnetotherapy and widely used in the treatment of patients with such diseases as epilepsy and rheumatoid arthritis, as well as in fracture and wound healing (Ciejka et al. 2011).

Our experiments have shown that male crickets exposed to an electromagnetic field generate calling songs with an increased chirp rate and shortened chirp period (by 2.7% and 5% respectively) compared to the control group but without the dominant sound frequency change. The observed effects may be related to the direct effects of the EMF on the neuromuscular system (e.g., motor neurons). Another explanation is an indirect or parallel EMF action through a stress reaction (increased motor activity and/or changes in stress-related hormone levels).

Given the current knowledge of electric and magnetic phenomena and their biological effects, it is possible that the central nervous system and sensory processes could be affected by EMF exposure, resulting in the changes in forewing movement frequency observed here. Observations supporting this assumption include (1) the effect of exposure to EMF (50 Hz, 7 mT) on neural circuits controlling appendage movement and muscular force in locusts (Wyszkowska et al. 2016), EMF (50 Hz, 1 mT), (2) the influence of EMF on Ca^2+^ channel expression in neuronal synapses leading to changes in neuronal activity (Sun et al. 2016), (3) a significant change in the Ca^2+^ influx after EMF (50 Hz, 50 µT) exposure (Barbier et al. 1996), and (4) changes in the cell membrane potential and distribution of ions due to EMF exposure, e.g., through the modification of the Na^+^/K^+^ ATPase activity, as the frequency of the enzyme turnover rate is close to 50 Hz (Blank 2005). Shepherd et al. (2021) observed a wing-beat frequency increase in locusts exposed to EMF (50 Hz, 1–7 mT) and suggested that the central pattern generator composed of interneurons and motor neurons, as well as mechanosensory signals, may be directly affected by EMF.

The male calling song informs a female about the species identity and location of a sexually mature male and enables the female to approach the male by phonotaxis. The treadmill setups (usually a free-spinning Styrofoam ball) are popular for long trials in phonotactic choice tests (Hiraguchi and Yamaguchi 2000). This setup allows to track the trajectory of a single individual in a very controlled environment. However, it monitors only one individual at a time, and insects are usually restrained, so they cannot jump, roll over, or accelerate suddenly (Guerra et al. 2010; Hedwig 2017).

Our results showed that the melody changed by the presence of EMF was more attractive than the natural signal to young (virgin) females but not to aged (3-week-old) females, which were attracted to both the changed and natural signals. The shorter chirp periods imply greater energetic investment per unit of time. Our findings are consistent with Wagner et al.’s (1995) conclusions that females prefer energetically costly male displays, which are likely to be produced by males in better condition, e.g., with higher pathogen resistance (Ryder and Siva-Jothy 2000). Moreover, females were found to prefer songs resembling those emitted by males during aggressive encounters (Pollack 1982). In several species, aggression is linked to reproductive success, as females prefer or are constrained to mate with dominant males because individuals in good condition (genetic/aerobic/energetic/body) can invest more energy into acoustic signaling and aggression (Bunting and Hedrick 2018). Perhaps this is why young females in our study selected the changed signal even though the natural call song was still not attractive to them.

The reproductive value should decline with age; thus, aged females should exhibit a higher motivation to mate and be less selective than younger females. In accordance with this assumption, we showed a lower selectivity of aged females with regard to signal quality. Prosser et al. (1997) revealed that older females of *Gryllus integer* (25–28 days after imaginal exclusion) exhibited greater movement towards a male calling song than younger females (11–14 days after imaginal exclusion). Aged females seemed more motivated to mate, while younger females appeared more selective, only exhibiting preferences in trials with multiple mate opportunities, which is in line with our findings. On the other hand, in our study, the tendency to approach the preferred (i.e., changed) signal was higher in 1-week-old females compared to older individuals (Fig. 6b), indicating that the young age group was particularly susceptible to environmental changes induced by EMF. The EMF-modified signal seems to be a stronger and more attractive stimulus for young cricket females compared to the natural calling sound of this species (acting more strongly and on younger females), suggesting a pre-existing bias in female preferences (Basolo 1990) and indicating potentially strong environmental effects of EMF pollution. It should be noted that, if exposure to EMF modifies mating signal parameters used by females to recognize the quality of signaling males, it may disrupt mate selection and switch the attraction of females towards suboptimal male individuals. Over a longer perspective, this may lead to a deterioration in the individual fitness of animals living in areas exposed to artificial electromagnetic fields.

Reactions to EMF (50 Hz, 1–7 mT) exposure as a stress factor can be observed as changes in behavior and physiology of insects leading to an increase in motor activity (Wyszkowska et al. 2006) and aggression level (Shepherd et al. 2019), impaired response to noxious heat (Maliszewska et al. 2018), reduced cognitive abilities (Shepherd et al. 2018), increase in stress protein levels (Wyszkowska et al. 2016), and enhanced oxidative stress response (Zhang et al. 2016). We have shown that male crickets responded to the exposure to EMF by increasing tyramine (by 65% relative to the control), serotonin (25%), and dopamine (50%) levels and reducing octopamine level (by 25%) in their brain. It is not clear whether these changes directly affected wing activity and modified calling song characteristics or are indicators of stress, which affected cricket behavior through some other mechanisms (e.g., the direct effect of EMF on motor neurons, see above). Moreover, the observed changes in the brains of insects exposed to EMF may indicate potential further physiological and behavioral consequences, finally leading to the deterioration of their functioning, fitness, and survival over a longer time scale. In mammals, dysfunctions in monoamine neurotransmission are implicated in neurological disorders, including Parkinson’s disease, schizophrenia, anxiety, and depression (Kobayashi 2001; Taylor et al. 2005). This also suggests insects as potential models to study the effects of EMF on humans.

Some studies have suggested that biogenic amine levels may be a potential area to elucidate the underlying mechanisms of EMF effects on insect behavior (Wyszkowska et al. 2006). Important processes in the regulation of motor behavior are initiated by the neuroendocrine system (Bunting and Hedrick 2018; Nässel and Winther 2010; Ohkawara and Aonuma 2016). Biogenic amines play a role in aggression, motivation, and mood as neurotransmitters, neuromodulators, and neurohormones in vertebrate and invertebrate nervous systems (Farooqui 2007; Ohkawara and Aonuma 2016; Roeder 2005; Roeder et al. 2003).

Octopamine (OA) and its biological precursor tyramine (TA) are the most frequent insect amines. Within the CNS, they act directly on the central pattern generator of flight and mating behavior, affect motivational states, and mediate aspects of aggression (Adamo et al. 1995; Hoyer et al. 2008; Matsumoto and Sakai 2001; Zhou et al. 2008). In the periphery, they sensitize sensory receptors, control neuromuscular transmission and muscle contraction kinetics, and enhance flight muscle glycolysis (Aonuma and Watanabe 2012; Brembs et al. 2007; Pflüger and Duch 2000; Roeder 2005; Szczuka et al. 2013; Vierk et al. 2009; Watanabe et al. 2011). Octopamine acts as a stress-responsive hormone and neuromodulator, which, under EMF exposure, can be rapidly released into the hemolymph, leading to a decrease in its concentration in the brain, as shown in our study. Increased TA level may be caused by external stimuli such as EMF and/or by OA depletion and activation of the synthesis mechanism. High levels of OA may also induce secondary effects including the desensitization of octopaminergic receptors or the reduction of endogenous octopamine release by autoregulation (Robertson and Juorio 1976).

Dopamine (DA) and serotonin (5-HT) are associated with motor control, arousal, and aggressive behaviors (Aonuma 2020; Dyakonova and Krushinsky 2013; Johnson et al. 2009; Kume et al. 2005; Stevenson et al. 2000). The increased dopamine level has been shown to drive high activity through cryptochrome (Kumar et al. 2012), whose role was also described in light-dependent magnetoreception in insects (Gegear et al. 2008; Netušil et al. 2021). DA was shown to be a key element in the response of *Drosophila* to metabolic, oxidative, and mechanical stressors (Neckameyer 1998). Moreover, DA was released in the honeybee brain after electric shock stimulation (Jarriault et al. 2018) and, together with 5-HT, in response to alarm pheromone, increasing the likelihood of stinging (Nouvian et al. 2018). Kume et al. (2005) confirmed the participation of DA in the regulation of insect arousal (hyperactivity and shortening of the rest phase). Chen et al. (2008), on the other hand, showed depressed brain OA levels (similar to our study) and DA levels, as well as an unchanged 5-HT level in bees exposed to stress. In light of the above-cited results, the increase in dopamine and serotonin levels in the cricket brain observed in our study suggests a stress-related response of these insects to EMF.

The present work for the first time demonstrated changes in biogenic amine levels in the insect brain occurring following exposure to EMF (50 Hz, 7 mT). Previously, changes in levels of biogenic amines under the influence of extremely low frequency-EMF have been determined in rats. Exposure to EMF (60 Hz, 2 mT) produced a significant increase in the levels of 5-HT and DA but a decrease in norepinephrine (a functional analog of OA in vertebrates) in the rat brain (Chung et al. 2014), which is consistent with our observations. Similarly, EMF (10 Hz, 1.8–3.8 mT, 1 h × 14 days) exposure increased the rate of synthesis (turnover) of DA and 5-HT in the rat frontal cortex (Sieroń et al. 2004). Another study showed that the affinity of serotonin 5-HT (2A) receptors decreased and their density increased in the prefrontal cortex of rats after EMF (50 Hz, 0.5 mT) exposure (Janać et al. 2009).

In insects, amines were measured after exposure to a static electric field (34–164 kV/m) (Newland et al. 2015), which reduced 5-HT and DA levels and increased OA level in the *Drosophila* brain. *Drosophila* avoided a static electric field, and the wings were involved in its detection. The observed behavior was related to the movement of electric charges on the surface of the insect body. However, such a mechanism is not expected during exposure to an alternating magnetic field, as in our study.

## Conclusions

The electromagnetic field (EMF, 50 Hz, 7 mT) exposure of the cricket *Gryllus bimaculatus* leads to behavioral and physiological changes. Findings indicate that changes in the chirp rate might be a stress-related behavior, as they are accompanied by changes in the levels of stress hormones in the brain.

The reproduction of crickets is important to the worlds of plants, animals, and humans. The cricket diet contains a lot of cellulose-rich plant materials. Bacteria and fungi easily decompose cricket fecal matter, increasing the energy and nutrient flows in the ecosystem. This provides plants with a rich source of easily available essential growth factors. Crickets also help control plant communities in both natural and human-made ecosystems. Additionally, they are essential food sources for insectivores (Rogers 2021). In our work, crickets were only a model indicating that such a phenomenon may be much more common in nature, including other insect species. Changes in calling songs induced by EMF exposure may confuse mating, which can lead to adverse health outcomes, alter population dynamics, and impair sexual selection. Ultimately, it may be of ecological importance unless adaptations to cope with anthropogenic disturbance appear in human-impacted populations (Bent et al. 2021). EMF is becoming a very strong and important environmental factor. Concerns should not be limited only to human health, but the ecological significance of EMF should also be considered. Our results lead to a call for further studies on the effects of anthropogenic electromagnetic field on insects (including pollinators and insect models), as well as the identification of key knowledge gaps in this field to improve our understanding of the effects of EMFs in the environment (Vanbergen et al. 2019).

## Supplementary Information

Below is the link to the electronic supplementary material.

Supplementary file1 (PDF 261 KB)

## Data Availability

The datasets used and/or analyzed during the current study are available from the corresponding author upon reasonable request.
