# Supplementary material for: Electromagnetic field exposure affects the calling song, phonotaxis, and level of biogenic amines in crickets
Source: Environ Sci Pollut Res Int. 2023 Jul 28;30(40):93255–68. doi: 10.1007/s11356-023-28981-0 (PMC10447283; doi:10.1007/s11356-023-28981-0)
Supplement: Supplementary file 1 — Supplementary file1 (PDF 261 KB) [file 11356_2023_28981_MOESM1_ESM.pdf]

Supplementary for:

Electromagnetic field exposure affects the calling song, phonotaxis, and level of biogenic amines in crickets.

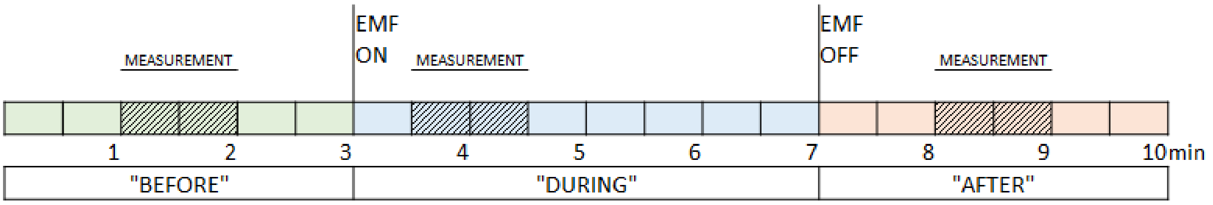

Fig. S1 Scheme of calling song recording. A single male was exposed in the chamber in the presence of a female. The male song was recorded for 3 min before the EMF was turned on, for 4 min during the exposure to the EMF and for another 3 min after the EMF was turned off (10 min of recoding altogether). Time windows for chirp measurements within each of these three periods are indicated

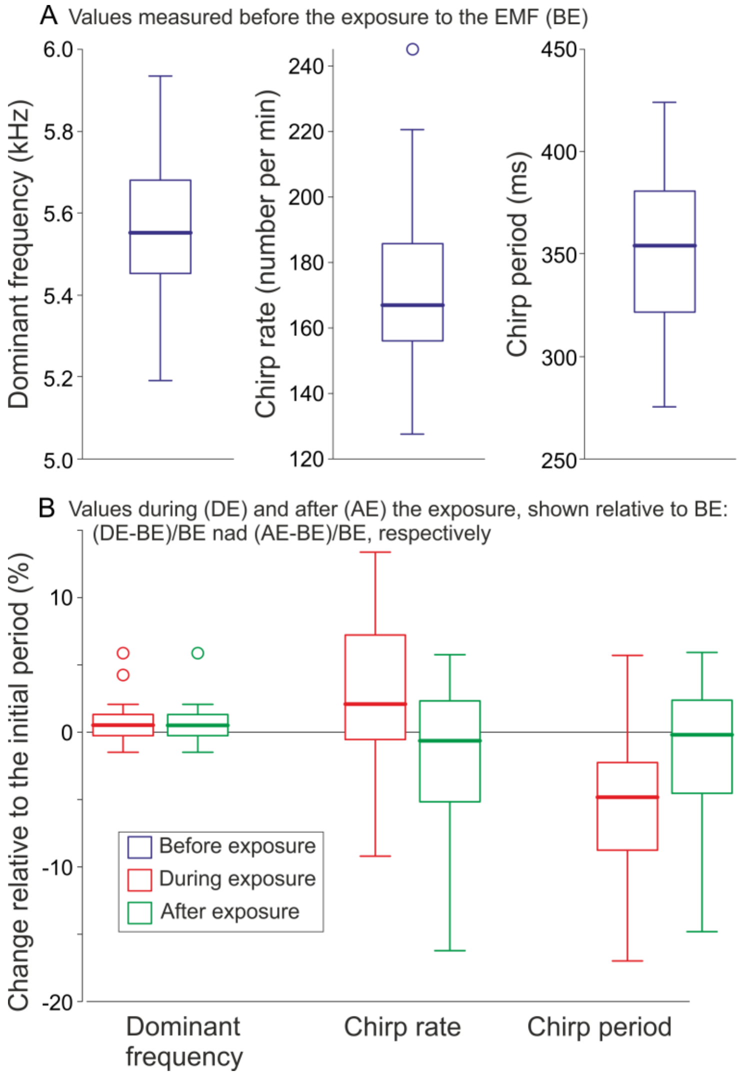

Fig. S2 Effect of the electromagnetic field on particular parameters of the male cricket calling song. (A) Initial parameters of the natural male cricket signal before their exposure (BE) to the electromagnetic field. (B) Changes observed in animals during (DE) and after the exposure (AE). The values in panel B are presented as percentage differences relative to the initial value ((DE-BE)/BE and (AE-BE)/BE, respectively). The boxplots present medians (horizontal lines), 1st-3rd quartiles (boxes), 1.5*interquartile range (whiskers) and outliers (circles). Values above and below zero indicate an increase and decrease in the parameter value, respectively

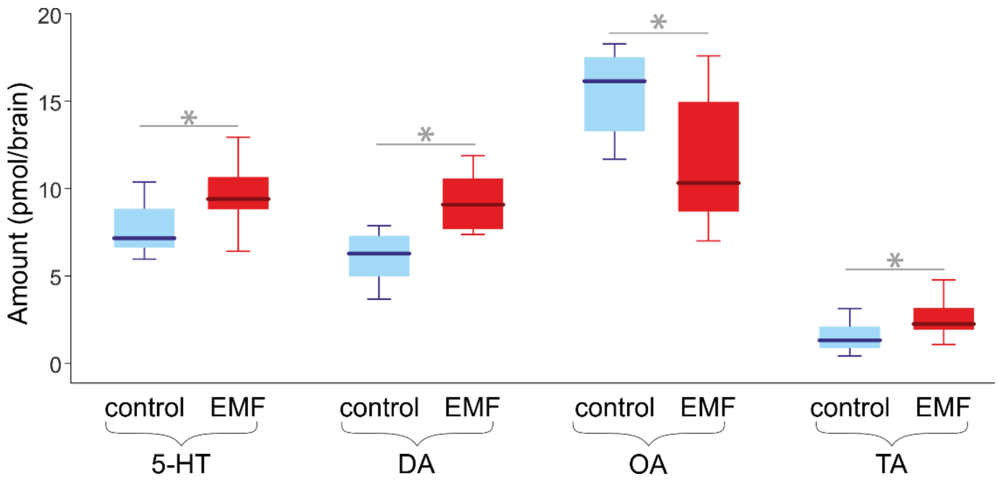

Fig. S3 Biogenic amine (serotonin: 5-HT, dopamine: DA, octopamine: OA, tyramine: TA) levels in the brains of male crickets exposed to electromagnetic field (EMF) and of control individuals. The boxplots present medians (horizontal lines), 1st-3rd quartiles (boxes), and 1.5*interquartile range (whiskers). Asterisks indicate significant effects of EMF (according to the analysis of principal components associated with particular amine levels)
